# Influence of Climate-Related Environmental Stresses on Economically Important Essential Oils of Mediterranean *Salvia* sp.

**DOI:** 10.3389/fpls.2022.864807

**Published:** 2022-05-04

**Authors:** Erna Karalija, Sabina Dahija, Petr Tarkowski, Sanja Ćavar Zeljković

**Affiliations:** ^1^Laboratory for Plant Physiology, Faculty of Science, University of Sarajevo, Sarajevo, Bosnia and Herzegovina; ^2^Centre of the Region Haná for Biotechnological and Agricultural Research, Czech Advanced Technology and Research Institute, Palacký University, Olomouc, Czechia; ^3^Centre of the Region Haná for Biotechnological and Agricultural Research, Department of Genetic Resources for Vegetables, Medicinal and Special Plants, Crop Research Institute, Olomouc, Czechia

**Keywords:** *Salvia* L., essential oil, bioactivity, environmental stress, chemodiversity

## Abstract

*Salvia* L. is the largest genus in the family Lamiaceae, with about 1,000 species and a nearly cosmopolitan distribution. *Salvia* species are used in both traditional and conventional medicines, and other numerous industries, such as spices and perfumes. The number of papers dealing with *Salvia* exceeds 12,000 and mostly investigates their chemical composition and bioactive properties. A smaller proportion of papers however consider environmental factors, mostly on the effects of microclimate conditions on its geographic distribution along an altitudinal or longitudinal gradient, and very few studies can be found on the effects of emerging stressors on the commercial production of sages of medicinal and economical importance. Here, we summarize available data on the essential oil composition of three economically important sages from the Mediterranean area, that is, *Salvia officinalis*, *Salvia officinalis* subsp. *lavandulifolia*, and *Salvia fruticosa*, and the effects of climate-related environmental stressors on their chemical profiles. Environmental stress factors, such as an increase in soil salinity and aridity, and changes in annual average temperatures, are going to impose a serious risk on the commercial production of sage essential oils, which are commercially produced in many European countries. This review highlights the already confirmed effects of these stressors on three selected *Salvia* species and consequently the importance of mitigating the effects of climate change on the commercial production of these essential oils.

## Introduction

The genus *Salvia* L. includes close to 1,000 species from all around the globe, and it is considered one of the largest genera in the Lamiaceae family. The diversification within the genus *Salvia* is considered to belong to biogeographical lines including three distinguished *Salvia* clades: clade I mostly distributed in the Old World and one lineage from the New World; clade II exclusively comprised of plants distributed in the New World; and, clade III which only includes the Asian lineage *Salvia* species (Walker et al., 2004). There are 36 *Salvia* species listed on European soil, including many endemic species mostly found in the Iberian and Balkan Peninsula with significant importance for perfumery, pharmaceutical, and other industries (Ardestani and Ghahfarrokhi, 2021). Some of the widely used *Salvia* species in traditional medicine are *Salvia officinalis* L., *Salvia fruticosa* Mill. (syn. *Salvia triloba* L.), *Salvia miltiorrhiza* Bunge, *Salvia hispanica* L., *Salvia sclarea* L., *Salvia triloba* L., *Salvia stenophylla* Burch. ex Benth., and *Salvia repens* Burch. ex Benth (Lopresti, 2016; Llurba-Montesino and Schmidt, 2018). However, in this review, we cover the selected *Salvia* species of European origin that possess the highest economical values and which are thought to be under the influence of climate-related environmental stresses.

**Figure 1:**
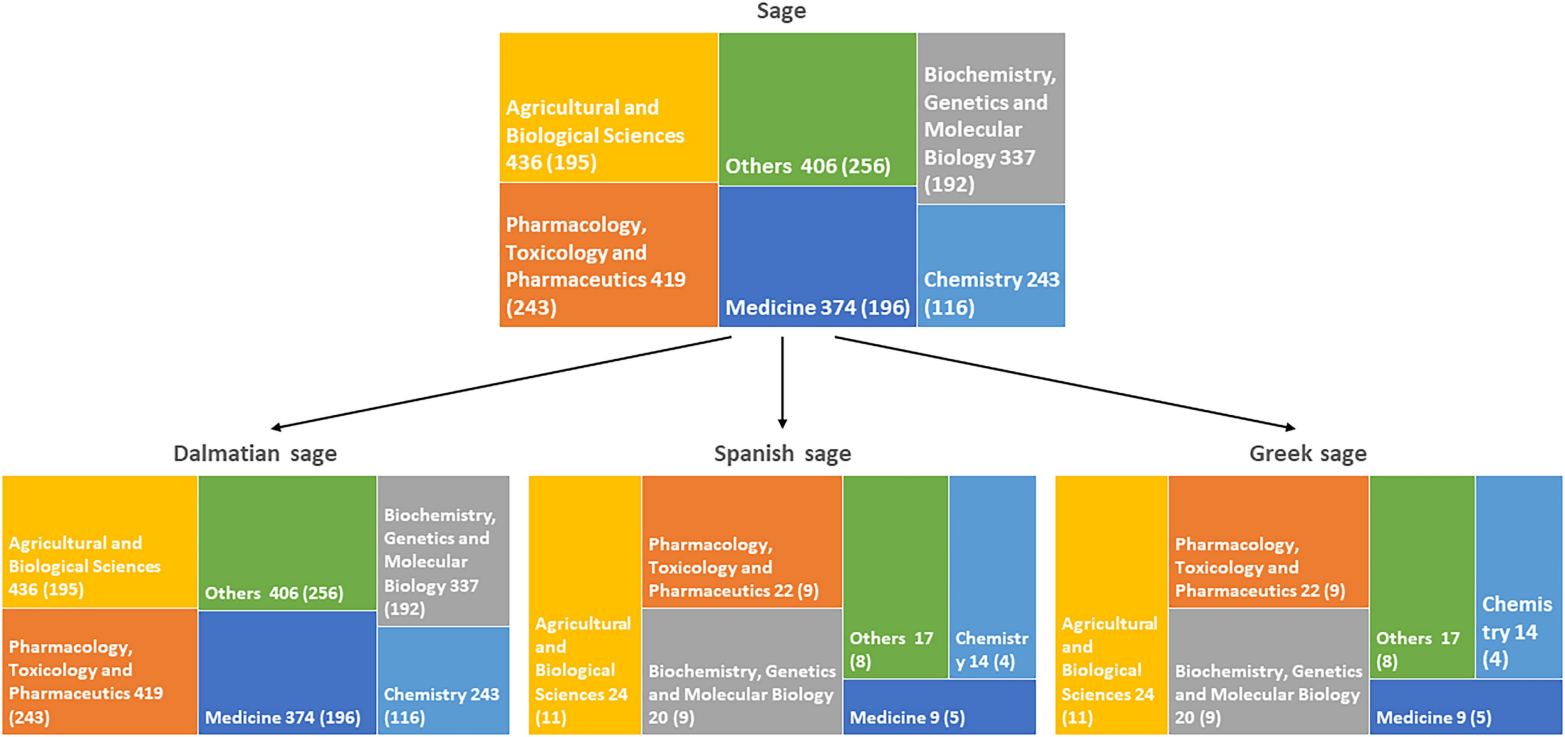
Numbers of studies by subject area on *Salvia* genus and species reviewed here. Numbers of studies since 2012 are indicated in the brackets.

Although species of *Salvia* L. are known since ancient times, plants belonging to this genus are still in scientific focus. The literature overview performed in Scopus® search engine shows over 12,000 reports on this genus, and more than half were published in the last 10 years. Most of the published reports are in the fields of medicine, pharmacology, and agricultural sciences (Figure 1). A large proportion of these studies (more than 1,000) was on essential oil composition, confirming that research on these complex mixtures is still important for their use in the pharmaceutical and food industries. The most investigated European species are *S. officinalis*, *S. officinalis* subsp. *lavandulifolia*, and *S. fruticosa*. Hence, we have decided to narrow this review to these three species.

There is a significant number of papers on the effects of environmental factors on *Salvia* species. Research shows that the composition and concentration of volatile compounds in *Salvia* are dependent not only on genetic and seasonal factors but also on environmental factors (Ramezani et al., 2020). Climate change is, with no doubt, one of the concerning, human-induced, environmental threats for plant biodiversity. The European Union is leading the way toward becoming the first climate-neutral zone by 2050 through the limitation of global warming, which was confirmed by the European Commission’s launch of the Green Deal in December 2019 (European Commission, 2019). Changes in the worlds’ climate could have serious impacts on the future distribution and industrial value of plants since an increase in global temperatures, disruption, and changes in hydroclimatic factors could lead to loss of habitats (Pressey et al., 2007).

## Essential Oils of *Salvia* L.

**Figure 2:**
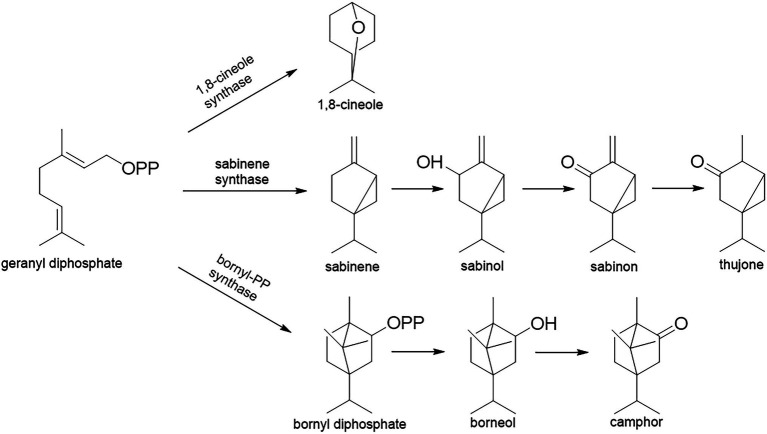
Monoterpene biosynthetic pathways in *Salvia* glandular trichomes.

The majority of *Salvia* species have a long tradition of use in folk medicine, but also as flavoring agents and ornamental plants [Code of Federal Regulation (21CFR172.510), 2021]. For example, *Salvia officinalis* L. and *S. fruticosa* Mill. are used as a mouth wash against the inflammations of the oral cavity. Terpenes from essential oils are the main carriers of the bioactivity of *Salvia* sp. They are synthesized in glandular hairs (trichomes), and they play a crucial role in the plants’ defense against phytophagous insects or pests (Giuliani et al., 2017). The essential oils of the *Salvia* species reviewed here mainly consist of cyclic oxygenated monoterpenes, that is, 1,8-cineole (eucalyptol), thujones (α- and β-), and camphor (Li et al., 2015). These compounds are synthesized *via* three different biosynthetic pathways, but all of them include cyclization of geranyl diphosphate (GPP; Figure 2): (i) 1,8-cineole *via* 1,8-cineole synthase (geranyl-diphosphate diphosphate-lyase; EC 4.2.3.108); (ii) thujones *via* sabinene synthase (geranyl-diphosphate diphosphate-lyase; EC 4.2.3.110), which firstly forms sabinene that is transformed to thujone *via* several steps which include the oxidative enzyme cytochrome P450, but also reductase; (iii) camphor *via* bornyl-PP-synthase [(+)-bornyl-diphosphate lyase; EC 5.5.1.8] that forms bornyl diphosphate which is then hydrolyzed and oxidized to camphor (Selmar and Kleinwächter, 2013; Radwan et al., 2017).

**Figure 3:**
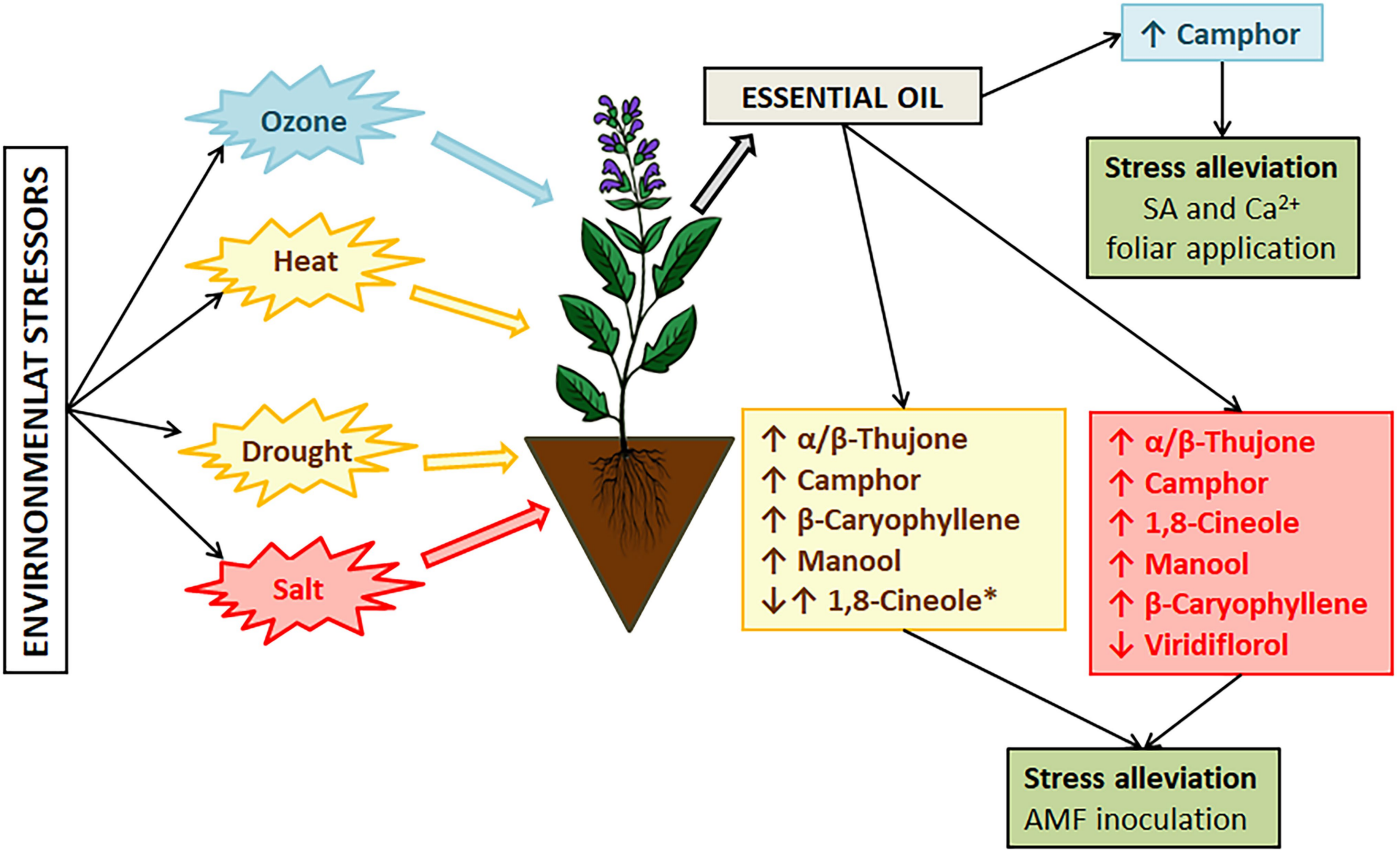
The major changes in sage essential oil composition induced by environmental stressors. ^*^Changes of 1,8-cineole levels also depends on sage species.

However, it is well known that the composition of essential oils strongly depends on environmental conditions, although with the accelerating climate change this can potentially be problematic for the future production and use of these oils in the pharmaceutical and food industry. The majority of studies on the essential oil composition of *Salvia* species are merely descriptive, that is, only dealing with the content percentage of the components (see references in Supplementary Table 1), but solid explanations about the differences in composition are lacking. Yet, few studies about the impact of abiotic stressors on terpene production can be found in the literature. According to Selmar and Kleinwächter (2013), drought stress induces stomatal closure and decreases CO_2_ uptake. As a result, plants respond by moving biosynthesis toward compounds that can reduce reactive oxygen species. In addition, Radwan et al. (2017) studied the metabolism of monoterpenes in *S. officinalis* grown under drought stress, and they found that enzymes (Figure 2) of stressed plants are upregulated, especially bornyl-PP synthase, resulting in higher levels of camphor produced (Figure 3). A common problem found in the *Salvia* literature lies in different methodologies used by the authors, for example, there is no consensus on the selection of the plant organ to be studied, its proper drying technique, homogenization, and even on the method used for identification of the constituents. Similar obstacles were pinpointed for the phytochemical analysis of selected *Mentha* species, also an economically important genus of the Lamiaceae family (Ćavar Zeljković et al., 2021b). Turek and Stintzing (2013) reviewed possible changes in terpene composition and factors affecting their stability, both in dried plant material or already isolated essential oil. In addition, Ćavar Zeljković et al. (2021a) showed that the content percentage of terpenes, calculated from the relative area of chromatographic peaks, cannot be a sufficient method for the chemical characterization of essential oils, especially if they are used in the food and pharmaceutical industries. They pointed out that levels of at least the major constituents should be presented in exact concentrations.

Despite the medicinal potential of *Salvia* essential oils being well established, precautions must be made since some of the major compounds of the oils are toxic to humans. For example, 1,8-cineole, essential oils of *Salvia* are often an ingredient in mouthwash solutions. This monoterpene epoxide has however low toxicity (De Vincenzi et al., 2002) and it is approved by the Food and Drug Administration for food use (CFR 172.510). But the toxicity of thujone has been extensively studied (Pelkonen et al., 2013). Thujone has antagonistic properties to γ-aminobutyric acid (GABA), which results in muscle spasms and convulsions (Höld et al., 2002). According to the European regulations, its levels should not exceed 25 mg/kg of food prepared from any of the sage species (Regulation EC, 2008). Although camphor is often an ingredient of topical medications used for muscle pain relief and a cold remedy for the relief of chest congestion (Valdez et al., 1999), its inhalation is very toxic. In high doses, this monoterpene ketone produced causes irritability, disorientation, lethargy, muscle spasms, vomiting, abdominal cramps, convulsions, and seizures, especially in children (Uc et al., 2000).

**Figure 4:**
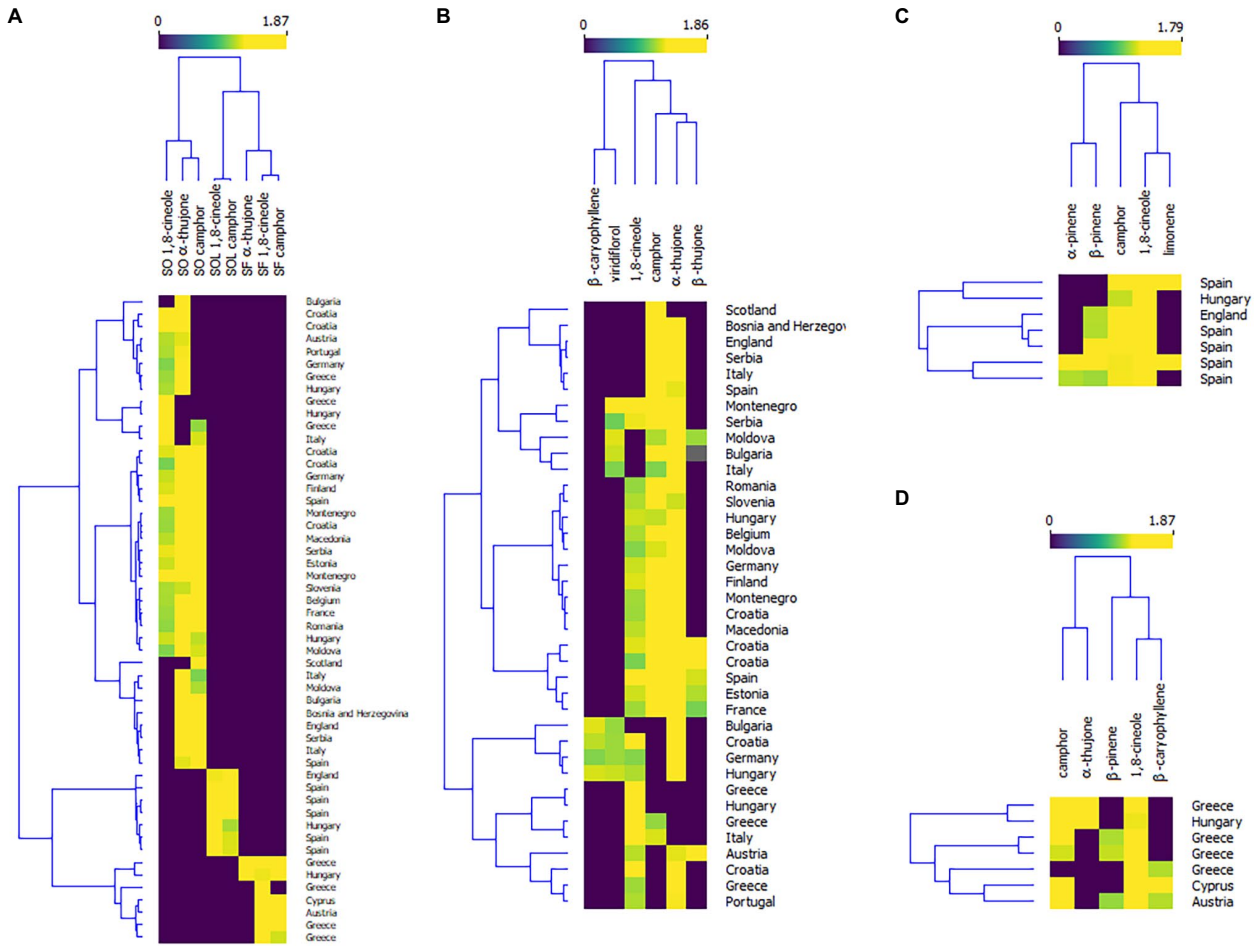
Heatmaps of the levels of the essential oil constituents in of *Salvia officinalis*—SO, *S. officinalis* subsp. *lavanulifolia*—SOL, and *S. fruticosa*—SF. **(A)** Country of origin in relation to the percentage content of the major monoterpenes in all three species; **(B)** Country of origin in relation to the percentage content of the major monoterpenes in *S. officinalis*; **(C)** Country of origin in relation to the percentage content of the major monoterpenes in, *S. officinalis* subsp. *lavanulifolia*; and **(D)** Country of origin in relation to the percentage content of the major monoterpenes in *S. fruticosa*.

Plant bioactive properties can be highly affected by environmental changes, such as changes in ozone concentrations, increased temperatures, changes in water regimes (drought and flooding), and an increase in soil salinity (Bettaieb et al., 2011). Therefore, the chemical composition of the oils of the mostly used *Salvia* species, Dalmatian sage (*Salvia officinalis*), and Spanish sage (*Salvia officinalis* subsp. *lavandulifolia*) are under the regulation of the International Standardization Organization (ISO). According to the ISO regulation, *S. officinalis* essential oil should contain 5.5%–13% of 1,8-cineole, 18%–43% of α-thujone, and 4.5%–45% of camphor (ISO 9909, 1997), while the oil of *S. officinalis* subsp. *lavandulifolia* should contain 10%–30% of 1,8-cineole, and 11%–36% of camphor (ISO 3526, 2005). Supplementary Table 1 summarizes the composition of the essential oils of the four *Salvia* species reviewed in this paper. The literature on *Salvia* species covers mainly the European continent (Figure 3). But wherever possible, we have reviewed whether these oils meet the ISO regulation. The heatmap in Figure 4A summarizes the literature data on levels of 1,8-cineole, camphor, and α-thujene (the main constituents of the oils of *S. officinalis*, *S. officinalis* susp. *lavandulifolia*, and *S. fruticosa*) from all three oils in different European countries. To construct the heatmap, the compounds with >10% were selected and their percentage content was log-transformed. Although it seems that the composition of these species is similar (Supplementary Table 1), they are separated into clusters. Monoterpenes from *S. officinalis* make one cluster, while monoterpenes from *S. offcinalis* subsp. *lavanulifolia* and *S. fruticosa* are in another cluster but grouped into two different subclusters.

## *Salvia officinalis* L.

Dalmatian sage or common sage (*Salvia officinalis* L.) from the Lamiaceae family is a well-known aromatic and medicinal Mediterranean plant (Avato et al., 2005; Raal et al., 2007). *Salvia officinalis* is native to the coastal regions of the western Balkan and southern Apennine Peninsulas with a habitat reaching south into northwest Greece (di Pietro, 2011). It is a perennial subshrub cultivated in temperate regions worldwide (Ghorbani and Esmaeilizadeh, 2017). Species of *Salvia* usually grow 30–70 cm tall, with a woody stem, whitish beneath and greyish-green above leaves, and with purple-blue flowers up to 3 cm long appearing from early summer to early autumn.

The use of the Dalmatian sage in traditional medicine has a long history with many studies even in recent years providing evidence for beneficial properties of this plant. Experimental studies on *S. officinalis* extracts and essential oils have revealed health-beneficial properties, such as antioxidant (Hohmann et al., 1999), antibacterial, hypoglycemic (Alarcon-Aguilar et al., 2002), anti-inflammatory (Ehrnhöfer-Ressler et al., 2013; Schröder et al., 2013), fungistatic, astringent, eupeptic, and anti-hydrolytic activity (Martins et al., 2015), hypotensive properties, central nervous system depressant actions, and anti-spasmodic activity. Additionally, the essential oil demonstrated bactericidal and bacteriostatic effects against both Gram-positive and Gram-negative bacteria (Mitić-Ćulafić et al., 2005) and reduced UV-induced mutations in *Escherichia coli* and *Saccharomyces cerevisiae* (Vuković-Gačić et al., 2006).

This review paper covers studies on *S. officinalis* (Dalmatian sage) essential oils from the European continent, with Croatian species as the most investigated (Lamien-Meda et al., 2010; Jug-Dujaković et al., 2012; Cvetkovikj et al., 2015). For better visualization, we have constructed a heatmap of the levels of the main essential oil constituents found in the available literature (Figure 4B). When we compared the profile of the major compounds, there was no meaningful clustering on the plants’ geographical origin. Although the essential oil composition varies even within a single country and it is often correlated with the microclimate conditions suggesting that the composition and yield are sensitive to even small disruptions in environmental conditions, emphasizing the significance of tracking the climate-related environmental stressors and their effect on plants.

The essential oil composition of Dalmatian sage from Croatia significantly differs from study to study. This plant naturally grows in a relatively small area (12,158 km^2^) but with different climatic conditions within the area. It is well known that the average temperatures and humidity significantly differ between the coastal area and continental area of Dalmatia. Generally, the contents of 1,8-cineole and camphor in the oil of Croatian sage are relatively similar in all published studies, while the levels of thujones are significantly different within the species (Supplementary Table 1), which is the reason why some of the oils from Croatian *S. officinalis* do meet the ISO legislation (ISO 9909, 1997). Again, the composition of the Dalmatian sage oil from Montenegro showed high variability in thujone content, while 1,8-cineole remains quite similar (Perry et al., 1999; Couladis et al., 2002; Damjanovic-Vratnica et al., 2008; Steševic et al., 2014). Also, studies of *S. officinalis* from Greece show significant intraspecific variability in the essential oil composition (Lamien-Meda et al., 2010). Interestingly, the authors detected significantly high levels of 1,8-cineole in *Greek S. officinalis* (Raal et al., 2007; Figure 4B).

*Salvia officinalis* is a drought-susceptible species (Munné-Bosch et al., 2001). In Mediterranean areas, the crop is subjected to summer drought stress, high temperature, soil salinity, and enhanced ozone levels (Pellegrini et al., 2018), which affect its composition and potential use. An increase in global temperatures is imposing additional stress on sage affecting essential oil composition and affecting sage cultivation and production due to leaf etiolation, wilting, and reduced photosynthetic capacity of plants (Lin et al., 2021). Under hot and arid conditions, accumulation of thujone has been recorded as a response to heat and water stress, while under optimal conditions camphor is mainly accumulated (Nevkrytaya et al., 2021). However, the results of Radwan et al. (2017) suggest that bornyl and sabinene synthases are upregulated by drought stress, while cineole synthase is downregulated by drought stress (Figure 3). Therefore, as also suggested by these authors, further research is needed.

The search for protective mechanisms to keep the oil composition stable and plants’ health has been ongoing during the past few years. Salicylic acid and calcium are recognized as signaling molecules in plants’ responses to oxidative stress induced by different environmental factors (Laanemets et al., 2013; Janda and Ruelland, 2015). Growth inhibition can be effectively alleviated by external application of salicylic acid (SA) or calcium (Ca^2+^) alone or in combination, as reported by Lin et al. (2021). They recorded an increase in new leaves formation under high-temperature stress when plants were treated with SA and Ca^2+^ (Figure 3).

Soil salinity is increasing due to progressive climate aridity (Taarit et al., 2009). Just in Tunisia alone, more than 10% of soil is affected by salinity consequently affecting sage growth and oil composition. In their research, they investigated how salinity levels can affect the chemical profile of *S. officinalis* and demonstrated that changes in the composition of essential oils correlate with the salinity level. Viridiflorol was the main component in the essential oils of plants that were not subjected to salinity stress. When plants were subjected to low salinity (25 mM NaCl) the main compound was the same, but it was represented in higher percentage (21.8%), moderate salt stress (50 and 100 mM NaCl) induced changes in compound synthesis resulting in 1,8-cineole (21.6 and 23.8% respectively) as the main oil compound followed by α-thujone (21.4 and 22.2%, respectively; Figure 3). Further increase in soil salinity induced higher synthesis of manool (65.7%). Moderate and severe salt stress induced a significant decrease of viridiflorol (13.4, 6.0, and 7.0% respectively). The accumulation of manool, labdane type diterpene, at high salinity levels can be exploited as a precursor of ambergris fragrant products (Taarit et al., 2010), but the severity of salt stress affects the physiological traits and yield, thus this can be considered more as a marker of severe salt stress in *S. offciinalis*. Alleviation of salt stress in sage has been confirmed after foliar application of SA with a variety of changes in the chemical composition of essential oils (Zohra Es-Sbihi et al., 2021). Sage essential oil from aerial plant parts is mainly composed of 1,8-cineole, α/β-thujone, and camphor, and salt stress can induce a significant increase in these compounds with some new compounds recorded, such as viridiflorol, β-caryophyllene, myrentol, and pulegone. When SA spray was applied water stress effects on major compounds were alleviated (Zohra Es-Sbihi et al., 2021). The application of SA can stimulate the conversion of thymol to carvacrol, and it can be correlated further with the synthesis of phytoalexins affecting the plants’ c potential (Sirvent and Gibson, 2002).

The occurrence of ozone in the troposphere because of NOx and volatile organic compounds emissions is considered to become an even bigger problem with climatic changes and anthropogenic activities. Ozone-induced injuries to plants include reduction of growth, photosynthetic rate, cell dehydration, and leaf necrosis (Sirvent and Gibson, 2002). Short exposure to ozone can be used for the enhancement of secondary metabolite production, especially to utilize excess excitation energy created by ozone exposure (Marchica et al., 2019). The main source of oxidative stress in plants exposed to drought lies in outbursts of toxic O_2_^−^ radicals because of electron overflow from the photosynthetic electron transport chains (Wilhelm and Selmar, 2011). Metabolic changes under oxidative stress result in large consumption of NADPH^+^ through biosynthesis of specialized metabolites, such as phenols, terpenoids, alkaloids, glucosinolates, and others affecting essential oil composition (Selmar and Kleinwächter, 2013). Changes in monoterpene content can be correlated to upregulated expression of monoterpene synthase and bornyl diphosphate synthase (Radwan et al., 2017). Drought stress affects different growing stages of sage and the associated increase in production of monoterpenes under water deficit is a result of upregulation of 1,8-cineole synthase and bornyl diphosphate synthase in different growth stages of sage resulting in increased production of 1,8-cineole and camphor (Ramezani et al., 2020; Figure 3). In the context of reoccurring stress in *S. officinalis* increase in α-pinene has been recorded under repetitive drought stress (Kulak, 2020). Contrasting results have been recorded as well where reduction of α-pinene is recorded under water deficit conditions (Rioba et al., 2015; Chrysargyris et al., 2018) but in these cases water deficit was acute and no further investigation regarding stress memory was performed. Such results suggest that stress memory in *S. officinalis* can be reflected in essential oil composition and environmental changes can have long-lasting effects that right now cannot be comprehended.

## *Salvia officinalis* subsp. *lavandulifolia* (Vahl) Gams

*Salvia officinalis* subsp. *lavandulifolia* Vahl., known as “Spanish sage,” is a native of the Iberian Peninsula. It is a small woody herbaceous perennial shrub up to 17–100 cm with mauve-blue flowers. It occurs preferably on the sandy-calcareous soils (at 350–2,000 m) of Spain, southeast of France, and northwest of Africa (Sáez, 2010). This plant is characterized by a small subshrub or herb up to 17–100 cm, with branched stems, and tector hairs. The leaves are elliptic to linear-lanceolate. The inflorescence is simple or branched, with 2–8 flowers. The flowers are regular, tubular, or campanulate, usually green or violet-purple, pubescent. The blooming develops for about 1 month in late spring and early summer (Sáez, 2010). The leaf presents seasonal dimorphism (Palacio and Montserrat-Marti, 2006). *Salvia lavandulifolia* found its use in Mediterranean folk medicine as spasmolytic, antiseptic, analgesic, sedative, and antioxidant (Porres-Martínez et al., 2013). Studies have shown the potential value of Spanish sage in dementia therapy attributed to its sedative, antioxidant, anti-inflammatory, estrogenic, and antimicrobial and anti-cholinesterase activities (Perry et al., 2002; Cutillas et al., 2017). The essential oil of this species has been described as a safe memory enhancer, suggesting it could be used in Alzheimer’s disease therapy (Kennedy et al., 2011).

The majority of essential oil compositions of Spanish sage published to date (Supplementary Table 1) meet the ISO legislative (ISO 3526, 2005). Most of the studies are performed in Spain, where this plant originates from Figure 4C. However, the oil of this species also shows high intraspecific variability, which depends on locality (Herraiz-Peñalver et al., 2010; Usano-Alemany et al., 2014), but also phenological stage (Porres-Martínez et al., 2014; Usano-Alemany et al., 2014; Méndez-Tovar et al., 2016). Generally, wild Spanish sages grown in southern and central Spain contain high levels of 1,8-cineole, while those grown on the north contain more camphor and its biosynthetic precursor borneol (Herraiz-Peñalver et al., 2010; Usano-Alemany et al., 2014). Also, it is described that the levels of 1,8-cineole in the oil are the highest after the flowering period, with contents remaining high until the formation of new young leaves (Usano-Alemany et al., 2014). In addition, Porres-Martínez et al. (2014) found the levels of both 1,8-cineole and camphor increase from the vegetative stage to the full flowering stage.

Temperature stress in *S. officinalis* subsp. *lavandulifolia* can affect the ratio of monoterpene and sesquiterpene fraction in essential oil (Usano-Alemany et al., 2014) resulting in different quality and effectiveness, and oils of different biological properties. Under elevated growing temperature above 24°C (up to 32°C), there is an observed significant decrease in monoterpenes and an increase in sesquiterpenes with changes in levels of β-caryophyllene, caryophyllene oxide, and manool (Figure 3). The recorded changes resulted in changes in chemotype. In the context of drought stress, Spanish sage is tolerant to moderate drought stress with consistent essential oil quality and yield as demonstrated in recent studies (García-Caparrós et al., 2019). During cultivation, significant deviation/change in the chemical composition of the oils can arise because of changing environmental conditions, such as an increase in temperature and decreased precipitation. In cases of mild arid conditions with higher temperatures, this species can produce more essential oils with variable proportions of compounds as recorded through a 4-year analysis of cultivated plants, that is, 1,8-cineole become a prominent compound through the years (Usano-Alemany et al., 2016).

## *Salvia fruticosa* Mill.

This shrub, with the former name *Salvia triloba* L., is also known as Greek sage. It is native to the Mediterranean including Southern Italy, southern parts of the Balkan Peninsula (Greece) to West Syria. Three-lobed sage is found on dry rocky limestone soils or the edges of pine forests, riverbeds, and roadsides, at altitudes from 100 to 800 m a.s.l. (Radosavljević et al., 2012). This plant is also cultivated as an ornamental plant in other Mediterranean countries (Papafotiou et al., 2021). It has been used in folk medicine since ancient times with many studies demonstrating antioxidant (Triantaphyllou et al., 2001), anti-inflammatory (El-Sayed et al., 2006), anti-cholinesterase (Şenol et al., 2011), and antifungal activity (Exarchou et al., 2002). Traditionally, it is used as a medicinal (herbal tea), culinary, and as a melliferous plant (Clebsch, 2003). Antibacterial and antifungal activity has been confirmed against food contaminants (Delamare et al., 2007) and soilborne pathogens (Pitarokili et al., 2002). In nature, this plant is very drought resistant but in cultivation, such as cultivation on green roofs for its melliferous properties it cannot tolerate drought (Papafotiou et al., 2021).

The essential oil of Greek sage has a long tradition of medicinal uses in Greece (Clebsch, 2003), but still is not included in ISO legislation. According to the studies summarized in Supplementary Table 1, this species showed even higher intraspecific variability than *S. officinalis*. As presented in Figure 4D, clustering is very weak, that is, there is no grouping of this oil according to its geographical origin. In most of the studies, the oil of this species does not contain significant levels of thujones, except in the studies of Karousou et al. (1998) and Máthé et al. (2010). On the other hand, the amounts of 1,8-cineole are around 50%–70% (Bellomaria et al., 1992; Giweli et al., 2013; Sarrou et al., 2016), but can be up to 83.5% (Zgheib et al., 2019). But Karioti et al. (2003) and Arikat et al. (2004) have shown that this species produces lower amounts of 1,8-cineole when plants are grown *in vitro*.

Improvement of stress tolerance and bioactive properties has been tested through arbuscular mycorrhizal fungi (AMF) inoculation of *S. fruticosa* and improvement of plant growth mass and chlorophyll content has been recorded. Significant improvement (33%) in the utilization of adsorbed energy in photochemistry was observed for plants with inoculation with *Rhizophagus irregularis* (Moustakas et al., 2020). AMF beneficial effects on *Salvia* sp. stress tolerance have been recorded in other cases as well, such as *Salvia hispanica* when inoculated with an inoculum containing *Glomus mosseae* spores, hyphae, and colonized maize roots (Ouzounidou et al., 2015; Figure 3).

## Conclusion

Plants are plastic organisms and respond to changes in the environment by adapting their metabolism to offset stress-induced damages. *Salvia* sp. as economically important plants are under direct environmental stress due to climate change, which affects their chemical composition influencing the quality and yield of essential oils and therefore can have serious economic consequences. This review emphasizes the already confirmed effects of environmental stressors on three selected *Salvia* sp. and at the same time highlights the importance of such research to mitigate the effects of stress factors in the commercial production of these essential oils. The data from the literature indicate that the chemical composition of the *Salvia officinalis* essential oils is mostly affected by the soil salinity, while the increase in temperature affects the ratio between monoterpenes and sesquiterpenes in the essential oil of *S. officinalis* subs. *lavandulifolia*. Although there are no papers investigating the direct influence of environmental stressors on the oil composition of *S. fruticosa*, it was previously found that inoculation of AMF has a beneficial effect on the plant yield. This strategy might be useful for other *Salvia* species too, to alleviate the effects of environmental stressors. The fact that out of 12,000 results related to *Salvia* only a few could be found directly investigating climate-related environmental stressors emphasizes the need for further research to explain how these stressors affect economically produced *Salvia* essential oils. Evaluating data available on *Salvia officinalis* species essential oils it is clear that their commercial production will be affected by the changing climate. The presented review stresses the importance of studies oriented toward the investigation of how complex climate-related stressors can affect the commercial production of essential oils, and what are our next steps in the alleviation of these effects. In addition, *in vitro* culture or vertical farming under controlled conditions could be prospective solutions that can ensure sustainable production of plants and enable constant levels of metabolites of interest.

## Author Contributions

EK and SC: conceptualization, writing, and editing. SD: writing. PT: writing, editing, and reviewing. All authors contributed to the article and approved the submitted version.

## Funding

This work was funded by project No. RO0418 (Sustainable systems and technologies, improving crop production for a higher quality of production of food, feed, and raw materials, under conditions of changing climate), the Ministry of Agriculture, Czechia, and the project “Plants as a tool for sustainable global development” (registration number: CZ.02.1.01/0.0/0.0/16_019/0000827) within the program Research, Development, and Education (OP RDE).

## Conflict of Interest

The authors declare that the research was conducted in the absence of any commercial or financial relationships that could be construed as a potential conflict of interest.

## Publisher’s Note

All claims expressed in this article are solely those of the authors and do not necessarily represent those of their affiliated organizations, or those of the publisher, the editors and the reviewers. Any product that may be evaluated in this article, or claim that may be made by its manufacturer, is not guaranteed or endorsed by the publisher.
