# Supplementary material for: Influence of Climate-Related Environmental Stresses on Economically Important Essential Oils of Mediterranean Salvia sp
Source: Front Plant Sci. 2022 May 4;13:864807. doi: 10.3389/fpls.2022.864807 (PMC9114806; doi:10.3389/fpls.2022.864807)
Supplement: Supplementary file 1 [file Table_1.docx]

**Supplementary table 1.** Chemical variability of essential oils of the most important Mediterranean *Salvia* species.

| **Major compounds (> 10 %)** | **Country** | | **ISO** | **Reference** |
| --- | --- | --- | --- | --- |
| *Salvia officinalis* | | | | |
| β-pinene (9.3-14.5 %), α-thujone (13.2-16.1%), camphor (19.8-24.0 %) | | *in vitro* plant culture (Spain) | + | (Santos-Gomes and Fernandes-Ferreira, 2003) |
| 1,8-cineole (9.4-14.2 %), α/β-thujone (33.0-53.7 %), camphor (10.3-18.6 %), α-humulene (5.8-14.8 %) | | Germany | +/- | (Brieskorn and Melchior, 1969) |
| 1,8-cineole (22.1-44.8 %), camphor (12.5-15.3 %), β-pinene (3.0-10.4 %) | | Italy* | - | (Svoboda and Deans, 1992) |
| 1,8-cineole (23.0-58.4 %), camphor (6.2-14.7 %), β-pinene (6.7-22.3 %) | | Turkey* | - |  |
| camphor (11.6-22.0 %), thujone (7.0-25.3 %) | | England* | +/- |  |
| 1,8-cineole (10.4-11.7 %), camphor (15.2-27.4 %), thujone (19.8-28.8 %) | | Croatia* | + |  |
| 1,8-cineole (42.0 %) | | Greece | - |  |
| 1,8-cineole (4.6-12.0 %), α-thujone (12.0-55.2 %), camphor (4.7-23.1 %), α-humulene (3.4-11.6 %) | | Spain*, *** | +/- | (Santos-Gomes and Fernandes-Ferreira, 2001) |
| 1,8-cineole (6.9-18.5 %), α-thujone (8.5-25.4 %), camphor (0.2-21.1 %), viridiflorol (9.2-26.1 %) | | Montenegro* | +/- | (Couladis et al., 2002) |
| 1,8-cineole (6.4-16.7 %), α-thujone (19.5-19.9 %), camphor (9.7-24.8 %), viridiflorol (6.0-9.6 %) | | Serbia* | +/- |  |
| 1,8-cineole (12.8-15.5 %), α-thujone (31.8-36.8 %), camphor (15.8-24.7 %) | | Finland* | +/- | (Galambosi et al., 2002) |
| 1,8-cineole (4.1-21.1 %), α-thujone (33.9-73.12 %) | | Croatia*** | + | (Zutic et al., 2003) |
| 1,8-cineole (12.7 %), α-thujone (17.4 %), α-humulene (13.3 %) | | Portugal | + | (Lima et al., 2004) |
| 1,8-cineole (11.3-13.3 %), α-thujone (12.2-16.0 %), β-thujone (16.4-20.5 %), manool (6.6-11.0 %) | | Austria** | + | (Grassi et al., 2004) |
| α-thujone (57.0 %), β-thujone (15.0 %), viridiflorol (14.2 %) | | Croatia | - | (Maksimović et al., 2007) |
| 1,8-cineole (11.9 %), α-thujone (21.0 %), β-thujone (10.1 %), camphor (23.9 %) | | France | + | (Raal et al., 2007) |
| 1,8-cineole (14.6 %), α-thujone (18.6 %), camphor (13.7 %) | | Hungary | + |  |
| 1,8-cineole (12.6 %), α-thujone (19.6 %), camphor (19.2 %) | | Belgium | + |  |
| 1,8-cineole (45.3 %), camphor (11.3 %) | | Greece | - |  |
| camphor (29.8 %), borneol (11.8 %) | | Scotland | + |  |
| α-thujone (18.7 %), β-thujone (11.7 %), camphor (12.7 %), viridiflorol (15.7 %) | | Moldova | + |  |
| 1,8-cineole (5.3-14.6 %), α-thujone (15.2-26.6 %), β-thujone (5.2-12.9 %), camphor (16.4-20.0 %) | | Estonia* | +/- |  |
| 1,8-cineole (12.0 %), α-thujone (29.5 %), camphor (22.5 %) | | Montenegro | + | (Damjanovic-Vratnica et al., 2008) |
| α-thujone (24.9-27.4 %), β-caryophyllene (9.2-15.8 %), viridiflorol (9.3-11.5 %) | | Bulgaria | + | (Lamien-Meda et al., 2010) |
| 1,8-cineole (15.5-20.5 %), α-thujone (24.2-36.4 %), β-caryophyllene (1.5-13.5 %), virifdiflorol (4.4-11.8 %) | | Croatia | +/- |  |
| 1,8-cineole (9.4-10.6 %), α-thujone (4.5-36.9 %), β-caryophyllene (2.6-10.5 %), virifdiflorol (4.9-11.8 %) | | Germany | + |  |
| 1,8-cineole (10.5-11.9 %), α-thujone (22.3-23.3 %), α-humulene (12.5-15.1 %) | | Greece | + |  |
| 1,8-cineole (11.3-12.7 %), α-thujone (26.1-28.7 %), β-caryophyllene (13.2-15.0 %), virifdiflorol (12.4-14.3 %) | | Hungary | + |  |
| α-thujone (28.7-33.1 %), camphor (6.3-10.4 %), virifdiflorol (10.1-10.4 %), manool (9.5-10.4 %) | | Italy | + |  |
| α-thujone (36.0 %) | | Hungary | + | (Máthé et al., 2010) |
| 1,8-cineole (5.0-15.7 %), α-thujone (12.3-49.7 %), β-thujone (1.7-44.9 %), camphor (5.5-36.5 %) | | Croatia* | +/- | (Jug-Dujaković et al., 2012) |
| α-thujone (7.8-20.1 %), camphor (10.5-20.6 %), γ-muurolene (2.9-13.8 %), sclareol (6.7-23.1 %) | | Italy* | + | (Russo et al., 2013) |
| α-thujone (16.9-40.3 %), camphor (12.7-35.4 %), 1,8-cineole (6.4-12.1 %), β-thujone (1.5-10.3 %) | | Montenegro* | + | (Steševic et al., 2014) |
| 1,8-cineole (10.9-12.7 %), α-thujone (13.0-14.4 %), camphor (19.0-23.0 %) | | Slovenia* | + | (Cvetkovikj et al., 2015) |
| 1,8-cineole (8.0-10.1 %), α-thujone (10.3-49.7 %), β-thujone (1.5-27.7 %), camphor (5.2-36.5 %) | | Croatia* | +/- |  |
| α-thujone (7.1-24.6 %), camphor (10.1-38.0 %) | | Bosnia and Herzegovina* | + |  |
| 1,8-cineole (6.8-13.5 %), α-thujone (17.5-27.3 %), camphor (12.8-26.7 %) | | Macedonia* |  |  |
| α-thujone (23.4-26.8 %), camphor (14.9-19.6 %) | | Serbia* | + |  |
| 1,8-cineole (6.4-11.4 %), α-thujone (13.3-18.4 %), camphor (3.8-25.4 %) | | Romania* | + |  |
| 1,8-cineole (8.4-10.6 %), α-thujone (13.9-23.4 %), camphor (6.4-15.3 %) | | Moldova* | +/- |  |
| camphor (12.5-25.0 %) | | Bulgaria* | + |  |
| 1,8-cineole (5.6-20.1 %), α-thujone (29.6-49.7 %), β-thujone (3.8-14.89 %), camphor (14.0-25.4 %) | | Spain* | + | (Cutillas et al., 2017) |
| *Salvia officinalis* subsp. *lavandufolia* | | | | |
| camphor (27.0 %), 1,8-cineole (17.0 %), borneol (14.5 %), β-pinene (12.0 %), bornyl acetate (10.2 %) | | England | + | (Perry et al., 2002) |
| α-pinene (6.7-23.2 %), β-pinene (3.8-19.2 %), limonene (0.8-16.6 %), 1,8-cineole (6.4-34.5 %), camphor (0.0-15.4 %) | | Spain* | +/- | (Herraiz-Peñalver et al., 2010) |
| β-pinene (9.0-11.7 %), 1,8-cineole (13.5-31.9 %), camphor (14.4-23.9 %), *d*-terpineol (7.2-12.0 %), ledol (8.1-10.8 %) | | Spain | + | (Porres-Martínez et al., 2013) |
| 1,8-cineole (5.7-62.7 %), camphor (0.8-28.7 %), β-caryophyllene (0.6-24.0 %), limonen (2.2-25.2 %) | | Spain* | +/- | (Usano-Alemany et al., 2014) |
| β-pinene (7.5-10.9 %), α-pinene (9.8-11.8 %), 1,8-cineole (25.2-31.3 %), camphor (11.0-15.6 %), | | Spain*** | +/- | (Porres-Martínez et al., 2014) |
| camphene (1.0-10.0 %), β-pinene (5.1-19.8 %), 1,8-cineole (6.2-33.7 %), camphor (2.8-22.4 %), | | Spain*** | +/- | (Méndez-Tovar et al., 2016) |
| 1,8-cineole (47.0 %), camphor (12.6 %) | | Hungary | - | (Máthé et al., 2010) |
| *Salvia fruticosa* | | | | |
| 1,8-cineole (22.5-37.5 %), viridiflorol (7.2-37.6 %), 13-*epi*-manool (4.6-25.7 %), β-caryophyllene (0.3-13.0 %), α-humulene (4.7-10.2 %) | | *in vitro* plant culture (Greece) | n/a | (Karioti et al., 2003) |
| 1,8-cineole (4.0-67.5 %), camphor (5.7-44.5 %), β-caryophyllene (1.4-23.0 %) | | Cyprus* | n/a | (Bellomaria et al., 1992) |
| 1,8-cineole (42.0-74.4 %), camphor (0.9-25.6 %), β-caryophyllene (1.3-13.2 %), β-pinene (1.2-11.6 %) | | Austria** | n/a | (Länger et al., 1996) |
| 1,8-cineole (22.7-64.2 %), camphor (0.9-30.3 %), α-thujone (1.5-19.2 %), β-thujone (1.3-25.6 %) | | Greece*, *** | n/a | (Karousou et al., 1998) |
| camphor (22.6-23.1 %), α-pinene (8.7-12.7 %), borneol (0-12.6 %), 1,8-cineole (6.9-31.4 %) | | Greece | n/a | (Koliopoulos et al., 2010) |
| 1,8-cineole (44.7-58.4 %), β-pinene (5.8-14.1 %), camphor (1.3-14.9 %) | | Greece*** | n/a | (Sarrou et al., 2016) |
| camphor (26.0 %), α-thujone (21.4 %), 1,8-cineole (16.9 %) | | Hungary | n/a | (Máthé et al., 2010) |

* Different locations; ** different isolations, *** different phenological stages; + essential oil composition is within the ISO standard range; - essential oil composition is not within the ISO standard range; +/- some of the samples from the study are failing into ISO standard range, and some of them are not; n/a not applicable.

Bellomaria, B., Arnold, N., Valentini, G., and Arnold, H. J. (1992). Contribution to the study of the essential oils from three species of *Salvia* growing wild in the eastern mediterranean region. *J. Essent. Oil Res.* 4, 607–614. doi:10.1080/10412905.1992.9698143.

Brieskorn, C. H., and Melchior, J. (1969). Quantitative Änderung der Terpenoide im Keimling und Blatt von *Salvia officinalis* L. unter verschiedenen Bedingungen 21. Mitt. über Inhaltsstoffe von Salvia off. L. *Arch. Pharm. (Weinheim).* 302, 921–931. doi:10.1002/ardp.19693021206.

Couladis, M., Tzakou, O., Mimica-Dukić, N., Jančić, R., and Stojanović, D. (2002). Essential oil of *Salvia officinalis* L. from Serbia and Montenegro. *Flavour Fragr. J.* 17, 119–126. doi:10.1002/ffj.1065.

Cutillas, A.B., Carrasco, A., Martinez-Gutierrez, R., Tomas, V., and Tudela, J. (2017). Composition and antioxidant, antienzymatic and antimicrobial activities of volatile molecules from Spanish *Salvia lavandulifolia* (Vahl) essential oils. *Molecules* 22, 1382. doi:10.3390/molecules22081382.

Cvetkovikj, I., Stefkov, G., Karapandzova, M., Kulevanova, S., and Satovic, Z. (2015). Essential oils and chemical diversity of southeast european populations of *Salvia officinalis* L. *Chem. Biodivers.* 12, 1025–1039. doi:10.1002/cbdv.201400273.

Damjanovic-Vratnica, B., Ðakov, T., Šukovic, D., and Damjanovic, J. (2008). Chemical composition and antimicrobial activity of essential oil of wild-growing *Salvia officinalis* L. from Montenegro. *J. Essent. Oil Bear. Plant.* 11, 79–89. doi:10.1080/0972060X.2008.10643602.

Galambosi, B., Galambosi, Z., Pessala, R., Hupila, I., Aflatuni, A., Repcak, M., et al. (2002). Yield and quality of selected herb cultivars in Finland. *Acta Hortic.* 576, 139–149. doi:10.17660/ActaHortic.2002.576.21.

Grassi, P., Novak, J., Steinlesberger, H., and Franz, C. (2004). A direct liquid, non-equilibrium solid-phase micro-extraction application for analysing chemical variation of single peltate trichomes on leaves of *Salvia officinalis*. *Phytochem. Anal.* 15, 198–203. doi:10.1002/pca.769.

Herraiz-Peñalver, D., Usano-Alemany, J., Cuadrado, J., Jordan, M. J., Lax, V., Sotomayor, J. A., et al. (2010). Essential oil composition of wild populations of *Salvia lavandulifolia* Vahl. from Castilla-La Mancha (Spain). *Biochem. Syst. Ecol.* 38, 1224–1230. doi:10.1016/j.bse.2010.10.015.

Jug-Dujaković, M., Ristić, M., Pljevljakušić, D., Dajić-Stevanović, Z., Liber, Z., Hančević, K., et al. (2012). High diversity of indigenous populations of dalmatian sage (*Salvia officinalis* L.) in essential-oil composition. *Chem. Biodivers.* 9, 2309–2323. doi:10.1002/cbdv.201200131.

Karousou, R., Vokou, D., and Kokkini, S. (1998). Variation of *Salvia fruticosa* essential oils on the island of Crete (Greece). *Bot. Acta* 111, 250–254. doi:10.1111/j.1438-8677.1998.tb00705.x.

Koliopoulos, G., Pitarokili, D., Kioulos, E., Michaelakis, A., and Tzakou, O. (2010). Chemical composition and larvicidal evaluation of *Mentha*, *Salvia*, and *Melissa* essential oils against the West Nile virus mosquito Culex pipiens. *Parasitol. Res.* 107, 327–335. doi:10.1007/s00436-010-1865-3.

Lamien-Meda, A., Schmiderer, C., Lohwasser, U., Börner, A., Franz, C., and Novak, J. (2010). Variability of the essential oil composition in the sage collection of the Genebank Gatersleben: A new viridiflorol chemotype. *Flavour Fragr. J.* 25, 75–82. doi:10.1002/ffj.1969.

Länger, R., Mechtler, C., and Jurenitsch, J. (1996). Composition of the essential oils of commercial samples of *Salvia officinalis* L. and *S. fruticosa* Miller: A comparison of oils obtained by extraction and steam distillation. *Phytochem. Anal.* 7, 289–293. doi:10.1002/(SICI)1099-1565(199611)7:6<289::AID-PCA318>3.0.CO;2-7.

Lima, C. F., Carvalho, F., Fernandes, E., Bastos, M. L., Santos-Gomes, P. C., Fernandes-Ferreira, M., et al. (2004). Evaluation of toxic/protective effects of the essential oil of *Salvia officinalis* on freshly isolated rat hepatocytes. *Toxicol. Vitr.* 18, 457–465. doi:10.1016/j.tiv.2004.01.001.

Maksimović, M., Vidic, D., Miloš, M., Edita Šolić, M., Abadžić, S., and Siljak-Yakovlev, S. (2007). Effect of the environmental conditions on essential oil profile in two Dinaric *Salvia* species: *S. brachyodon* Vandas and *S. officinalis* L. *Biochem. Syst. Ecol.* 35, 473–478. doi:10.1016/j.bse.2007.02.005.

Máthé, I., Máthé, Á., Hohmann, J., and Janicsák, G. (2010). Volatile and some non-volatile chemical constituents of Mediterranean *Salvia* species beyond their native area. *Isr. J. Plant Sci.* 58, 273–277. doi:10.1560/IJPS.58.3-4.273.

Méndez-Tovar, I., Novak, J., Sponza, S., Herrero, B., and Asensio-S-Manzanera, M. C. (2016). Variability in essential oil composition of wild populations of Labiatae species collected in Spain. *Ind. Crop. Prod.* 79, 18–28. doi:10.1016/j.indcrop.2015.10.009.

Perry, N. S. L., Houghton, P. J., Jenner, P., Keith, A., and Perry, E. K. (2002). *Salvia lavandulaefoli*a essential oil inhibits cholinesterase in vivo. *Phytomedicine* 9, 48–51. doi:10.1078/0944-7113-00082.

Porres-Martínez, M., González-Burgos, E., Accame, M. E. C., and Gómez-Serranillos, M. P. (2013). Phytochemical composition, antioxidant and cytoprotective activities of essential oil of *Salvia lavandulifolia* Vahl. *Food Res. Int.* 54, 523–531. doi:10.1016/j.foodres.2013.07.029.

Porres-Martínez, M., González-Burgos, E., Carretero, M. E., and Gómez-Serranillos, M. P. (2014). Influence of phenological stage on chemical composition and antioxidant activity of *Salvia lavandulifolia* Vahl. essential oils. *Ind. Crops Prod.* 53, 71–77. doi:10.1016/j.indcrop.2013.12.024.

Raal, A., Orav, A., and Arak, E. (2007). Composition of the essential oil of *Salvia officinalis* L. from various European countries. *Nat. Prod. Res.* 21, 406–411. doi:10.1080/14786410500528478.

Russo, A., Formisano, C., Rigano, D., Senatore, F., Delfine, S., Cardile, V., et al. (2013). Chemical composition and anticancer activity of essential oils of Mediterranean sage (*Salvia officinalis* L.) grown in different environmental conditions. *Food Chem. Toxicol.* 55, 42–47. doi:10.1016/j.fct.2012.12.036.

Santos-Gomes, P. C., and Fernandes-Ferreira, M. (2001). Organ- and season-dependent variation in the essential oil composition of *Salvia officinalis* L. cultivated at two different sites. *J. Agric. Food Chem.* 49, 2908–2916. doi:10.1021/jf001102b.

Santos-Gomes, P. C., and Fernandes-Ferreira, M. (2003). Essential oils produced by in vitro shoots of sage (Salvia officinalis L.). *J. Agric. Food Chem.* 51, 2260–2266. doi:10.1021/jf020945v.

Sarrou, E., Martens, S., and Chatzopoulou, P. (2016). Metabolite profiling and antioxidative activity of Sage (*Salvia fruticosa* Mill.) under the influence of genotype and harvesting period. *Ind. Crops Prod.* 94, 240–250. doi:10.1016/j.indcrop.2016.08.022.

Steševic, D., Ristic, M., Nikolic, V., Nedovic, M., Cakovic, D., and Šatovic, Z. (2014). Chemotype diversity of indigenous dalmatian sage (*Salvia officinali*s L.) populations in Montenegro. *Chem. Biodivers.* 11, 101–114. doi:10.1002/cbdv.201300233.

Svoboda, K. P., and Deans, S. G. (1992). A study of the variability of Rosemary and Sage and their volatile oils on the British market: Their antioxidative properties. *Flavour Fragr. J.* 7, 81–87. doi:10.1002/ffj.2730070207.

Usano-Alemany, J., Palá-Paúl, J., and Herráiz-Peñalver, D. (2014). Comprehensive phenological description of essential-oil chemotypes of *Salvia lavandulifolia* VAHL grown under the same environmental conditions. *Chem. Biodivers.* 11, 1963–1977. doi:10.1002/cbdv.201400090.

Zutic, I., Putievsky, E., and Dudai, N. (2003). Influence of harvest dynamics and cut height on yield components of sage (*Salvia officinalis* L.). *J. Herbs Spices Med. Plants* 10, 49–61. doi:10.1300/J044v10n04_06.
